# The role of integrin-related genes in atherosclerosis complicated by abdominal aortic aneurysm

**DOI:** 10.1097/MD.0000000000040293

**Published:** 2024-11-15

**Authors:** Degao Hong, Likang Ma, Lei Jin, Lele Tang, Liangwan Chen, Zhihuang Qiu

**Affiliations:** a Department of Cardiology, Shanghang County Hospital, Longyan, Fujian, China; b Department of Cardiovascular Surgery, Fujian Medical University Union Hospital, Fuzhou, Fujian, China; c Key Laboratory of Cardio-Thoracic Surgery (Fujian Medical University), Fujian Province University, Fuzhou, Fujian, China.

**Keywords:** abdominal aortic aneurysm, atherosclerosis, bioinformatics, hub genes, microarray

## Abstract

Increasingly, the shared risk factors and pathological processes of atherosclerosis and abdominal aortic aneurysm (AAA) are being recognized. The aim of our study was to identify the hub genes involved in the pathogenesis of atherosclerosis and AAA. The analysis was based on 2 gene expression profiles for atherosclerosis (GSE28829) and AAA (GSE7084), downloaded from the Gene Expression Omnibus database. Common differential genes were identified and an enrichment analysis of differential genes was conducted, with construction of protein–protein interaction networks, and identification of common hub genes, and predicted transcription factors. The analysis identified 133 differentially expressed genes (116 upregulated and 17 downregulated), with the enrichment analysis identifying a potential important role of integrins and chemokines in the common immune and inflammatory responses of atherosclerosis and AAA. Regulation of the complement and coagulation cascades and regulation of the actin cytoskeleton were associated with both diseases, with 10 important hub genes identified: TYROBP, PTPRC, integrin subunit beta 2, ITGAM, PLEK, cathepsin S, lymphocyte antigen 86, ITGAX, CCL4, and FCER1G. Findings identified a common pathogenetic pathway between atherosclerosis and AAA, with integrin-related genes playing a significant role. The common pathways and hub genes identified provide new insights into the shared mechanisms of these 2 diseases and can contribute to identifying new therapeutic targets and predicting the therapeutic effect of biological agents.

## 1. Introduction

Abdominal aortic aneurysms (AAA) are considered to have occurred if the local diameter of the abdominal aorta exceeds 50% of the normal diameter and the dilation is irreversible.^[1]^ Atherosclerosis, characterized by abnormal vascular intima formation due to hyperlipidemia and lipid oxidation, may play a role in AAA formation. Fatty deposits of atherosclerotic plaques in the intima of arterial walls cause a proliferation of fibrous tissue and of the surrounding smooth muscles, leading to arterial stiffening.^[2]^ Moreover, both atherosclerosis and AAA share common risk factors, such as family history, male sex, advanced age, and smoking,^[3]^ and the pathological processes of chronic inflammation, extracellular matrix degradation, vascular smooth muscle apoptosis, and thrombosis are involved in both AAA and atherosclerotic plaque formation.^[4,5]^ Therefore, atherosclerosis may potentially promote AAA^[6,7]^ by causing a mechanical weakening of the aortic wall, loss of elasticity, and degenerative ischemic changes in the adventitial layer.^[8]^ However, the exact mechanism linking the pathogenesis of atherosclerosis and to AAA formation remains unclear. Identifying the common transcriptional signatures of atherosclerosis and AAA may clarify the shared pathogenetic pathway. Accordingly, our aim in this study was to identify the hub genes involved in the pathogenesis of atherosclerosis complicated by AAA.

## 2. Methods

### 2.1. Data source

The Gene Expression Omnibus (GEO) database (http://www.ncbi.nlm.nih.gov/geo) was used,^[9]^ with “atherosclerosis” and “abdominal aortic aneurysms or AAA” used as keywords to search the dataset for related genes. GEO is a public database containing a large number of high-throughput sequencing and microarray datasets, submitted by research institutes worldwide. For our study, we used the following 2 microarray datasets, GSE28829^[10]^ and GSE7084^[11]^. The GSE28829 dataset, created on the GPL570 (Affymetrix Human Genome U133 Plus 2.0 Array) platform, contains 16 advanced atherosclerotic plaque samples (thin or thick fibrous cap atheroma) (AA) and 13 early atherosclerotic plaque samples (intimal thickening and intimal xanthoma) as a control (CA), obtained from the human carotid artery. From the GSE7084 dataset, we chose the GPL2507 (Sentrix Human-6 Expression BeadChip) for a larger sample size, which contains 7 AAA samples and 8 control abdominal aorta samples (CO) obtained from autopsy.

### 2.2. Identification of differentially expressed genes

Comparison of the gene expression profile between the disease and control groups was performed using the GEO query R package (GEO2R; https://www.ncbi.nlm.nih.gov/geo/geo2r/?acc=GSE28829), to identify the differentially expressed genes (DEGs), and the Limma R package, to calculate multiple differential expressions.^[12]^ DEGs were identified by an adjusted *P* < .05 and an absolute fold-change (|logFC|)|≥|1. Probes that did not contain a corresponding gene were removed. If a gene corresponded to multiple probes, the 1 with the largest difference in expression was selected. The common set of DEGs between atherosclerosis and AAA was identified using a Venn diagram tool (http://bioinformatics.psb.ugent.be/webtools/Venn/).

### 2.3. Enrichment analyses of DEGs

Gene Ontology (GO) and Kyoto Encyclopedia of Genes and Genomes (KEGG) pathway enrichment analysis results for DEGs were obtained using the Database for Annotation, Visualization and Integrated Discovery (https://david.ncifcrf.gov/tools.jsp), which allowed us to investigate the biological functions and signaling pathways involved in a given gene set.^[13]^ GO includes 3 independent categories, namely biological processes, molecular functions, and cellular components. Terms with a *P* < .05 were considered significantly enriched.

### 2.4. Protein–protein interaction network construction and module analysis

The relationship between proteins of interest was obtained using the Search Tool for the Retrieval of Interacting Genes (STRING 11.5; https://cn.string-db.org/), which includes both direct binding relationships and coexisting upstream and downstream regulatory pathways.^[14]^ This information can be used to construct a protein–protein interaction (PPI) network with complex regulatory relationships; interactions having a combined score > 0.4 were considered significant. The PPI network was visualized using Cytoscape (Version 3.9.1 https://cytoscape.org/).^[15]^ The Cytoscape plug-in molecular complex detection technology was used to analyze the key functional modules, applying the following selection criteria: K-core = 2; degree cutoff = 2; maximum depth = 100; and node score cutoff = 0.2. The GO- and KEGG-based analyses of involved modular genes were then performed using Database for Annotation, Visualization and Integrated Discovery.

### 2.5. Selection and analysis of hub genes

Hub genes were identified using the CytoHubba plug-in (Cytoscape, version 3.9.1), with the following 8 common algorithms used to then evaluate and select hub genes: MCC, MNC, EPC, degree, closeness, radiality, bottleneck, and eccentricity. A co-expression network of these hub genes was then constructed using GeneMANIA (http://genemania.org/), a reliable tool for identifying internal associations within gene sets.^[16]^

### 2.6. Validation of hub genes expression in other datasets

The mRNA expression of the hub genes was validated using 2 additional datasets, GSE100927^[17]^ and GSE98278.^[18]^ Dataset GSE100927 includes 69 human samples of AA and 35 control artery samples (CA), while GSE98278 includes 31 human AAA samples and 17 peripheral normal aortic samples (CO) that collected during rupture repair of AAA for comparison. Comparison of the 2 datasets was performed using Student *t* test, with a *P* < .05 considered significant.

### 2.7. Prediction and verification of transcription factors

To more accurately predict the transcription factors (TFs) that regulate the hub genes, the following 2 databases were used: Transcriptional Regulatory Relationships Unraveled by Sentence-based Text mining (TRRUST) (https://www.grnpedia.org/trrust/) and ChIP-X enrichment analysis 3 (ChEA3) (https://maayanlab.cloud/chea3/). For the ChEA3 database, the ENCODE library was selected, with the significance level set at *P* < .05. The TRRUST database, used to predict transcriptional regulatory networks, contains the target genes corresponding to TFs and the regulatory relationships between TFs. The TRRUST database currently includes 2 species, humans and mice, with 8444 and 6552 TFs that target regulatory relationships of 800 human TFs and 828 mouse TFs, respectively.^[19]^ The ChEA3 database is a TF enrichment analysis tool that contains a collection of gene set libraries generated from multiple sources, including TF-gene co-expression from RNA-seq studies, TF-target associations from ChIP-seq experiments, TF-gene co-occurrence computed from crowd-submitted gene lists, and ranks TFs associated with user-submitted gene sets.^[20]^ In our study, common TFs predicted by both databases were selected. Finally, expression of TFs in datasets GSE28829 and GSE7084 was verified using Student *t* test.

## 3. Results

### 3.1. Identification of DEGs

**Figure 1.**
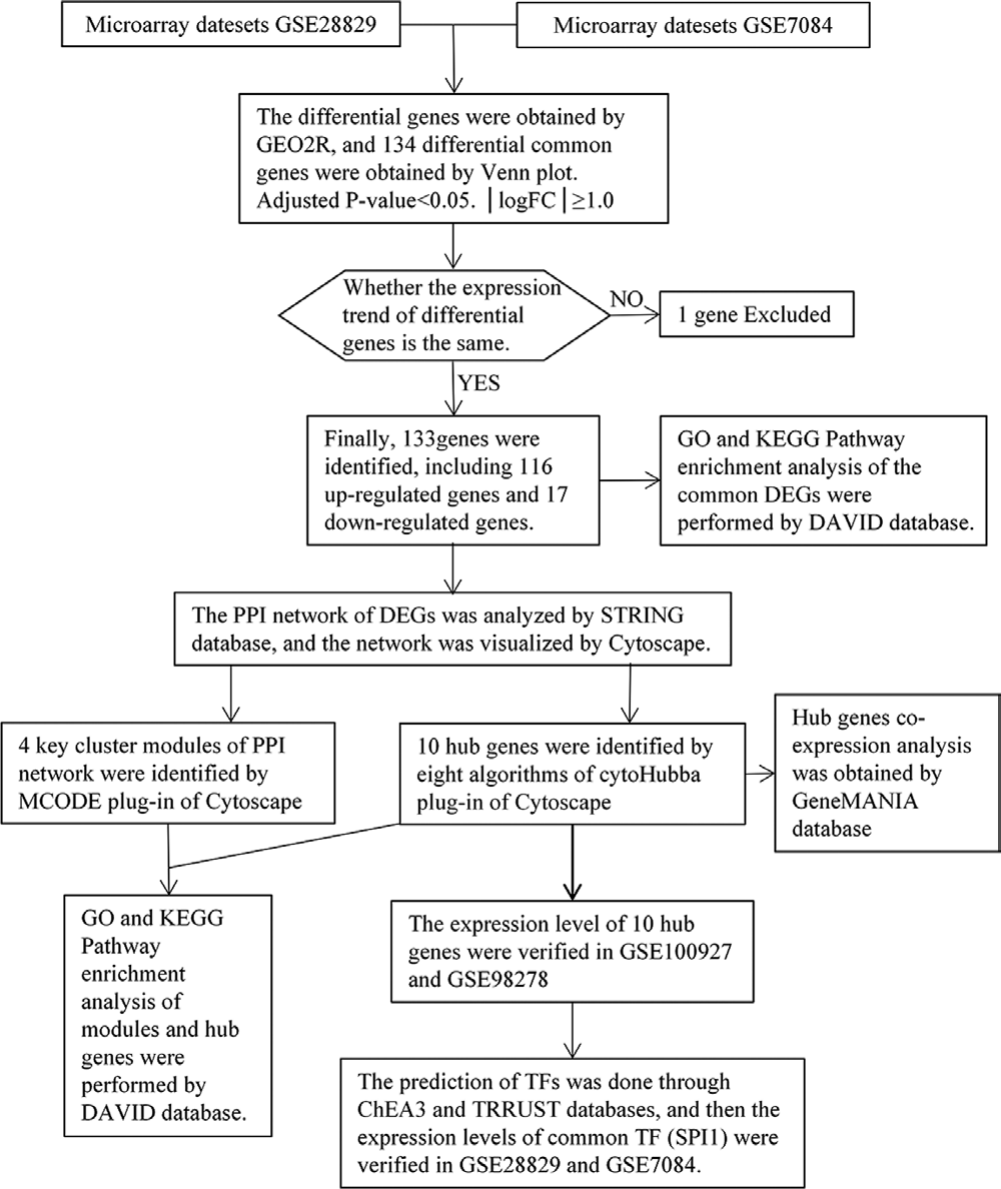
The study flow chart.

**Figure 2.**
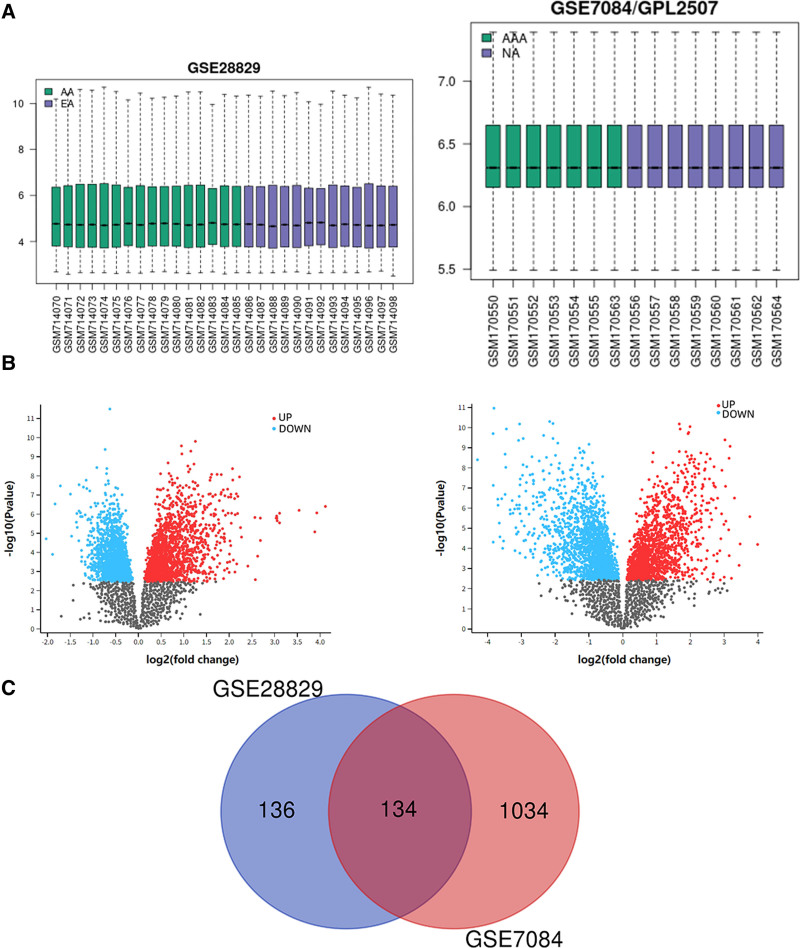
Identification of common differentially expressed genes (DEGs). (A) Box plot after normalization of the GSE28829 and GSE7084 datasets. (B) Volcano plot of GSE28829 and GSE7084, with red data points indicating upregulated genes and blue indicating downregulated genes. (C) Venn diagram of the GSE28829 and GSE7084 datasets, identifying an overlap of 134 DEGs.

The study process flow chart is shown in Figure 1. The data distribution, after standardization of the microarray data, is shown in Figure 2A. The volcano plots of DEGs (270 in GSE28829 and 1168 in GSE7084), obtained by difference analysis, are shown in Figure 2B. Using the Venn diagram, we identified 134 overlapping DEGs (Fig. 2C). Verification of these DEGs in the 2 datasets identified 133 DEGs with the same expression trend, including 116 upregulated and 17 downregulated genes (Table S1, Supplemental Digital Content, http://links.lww.com/MD/N877).

### 3.2. Analysis of the functional characteristics of common DEGs

**Figure 3.**
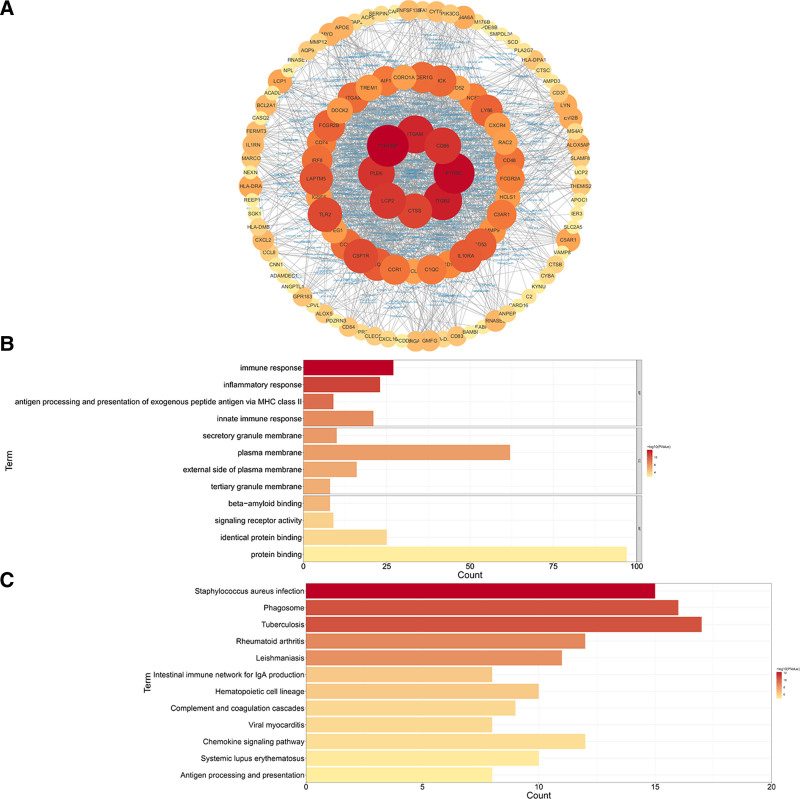
Protein–protein network (PPI) and the enrichment analysis of differentially expressed genes (DEGs). (A) The PPI network constructed by differential genes is shown; the darker the color, the higher the degree of interaction. (B) Results of the GO enrichment analysis of DEGs. (C) Results of the KEGG Pathway enrichment analysis of DEGs. GO = gene ontology; KEGG = Kyoto Encyclopedia of Genes and Genomes.

GO and KEGG pathway analyses were performed on the 133 DEGs to determine the associated biological process, cell component, and molecular function. Results of the GO analysis showed that DEGs were mainly enriched in the immune (*P* = 1.34E-16) and inflammatory (*P* = 3.19E-14) responses, antigen processing and presentation of exogenous peptide antigen via MHC class II (*P* = 1.48E-11), and the innate immune response (*P* = 2.25E-09) (Fig. 3B). In the KEGG pathway analysis, the following 3 significantly enriched pathways were identified: complement and coagulation cascades (*P* = 2.28E-06), chemokine signaling (*P* = 3.83E-06), and antigen processing and presentation (*P* = 1.40E-05) (Fig. 3C). These results illustrate the important roles of antigen processing and in immune inflammatory responses in both AAA and atherosclerosis.

### 3.3. PPI network construction and module analysis

**Figure 4.**
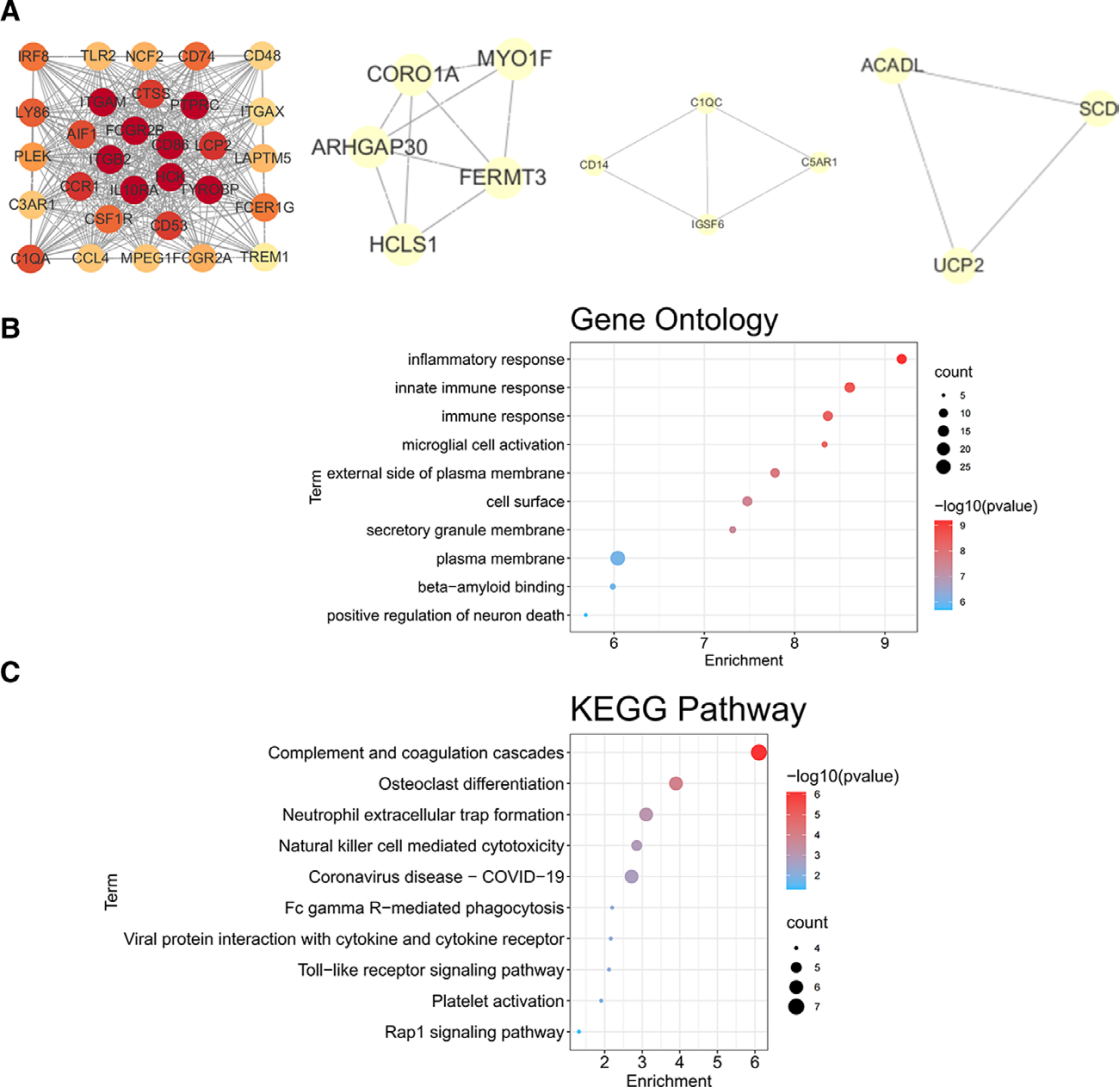
Gene enrichment analysis. (A) Four key gene modules obtained using the MCODE plug-in of Cytoscape. (B) GO enrichment analysis of the modular genes. (C) KEGG Pathway enrichment analysis of the modular genes. GO = gene ontology; KEGG = Kyoto Encyclopedia of Genes and Genomes; MCODE = molecular complex detection technology.

The PPI network of common DEGs, constructed in Cytoscape combining Search Tool for the Retrieval of Interacting Genes scores > 0.4, contained 116 nodes and 1197 edges (Fig. 3A). Four closely related gene modules, including 43 DEGs and 429 edges, were identified using the molecular complex detection technology plug-in of Cytoscape (Fig. 4A). The GO analysis revealed that these genes were related to inflammatory, innate immune, and immune responses (Fig. 4B). The KEGG pathway analysis showed that these genes were mainly involved in neutrophil extracellular trap formation, Fc gamma R-mediated phagocytosis, viral protein interaction with cytokines and the cytokine receptor, Toll-like receptor signaling, and the Rap1 signaling pathway (Fig. 4C).

### 3.4. Selection and analysis of hub genes

**Figure 5.**
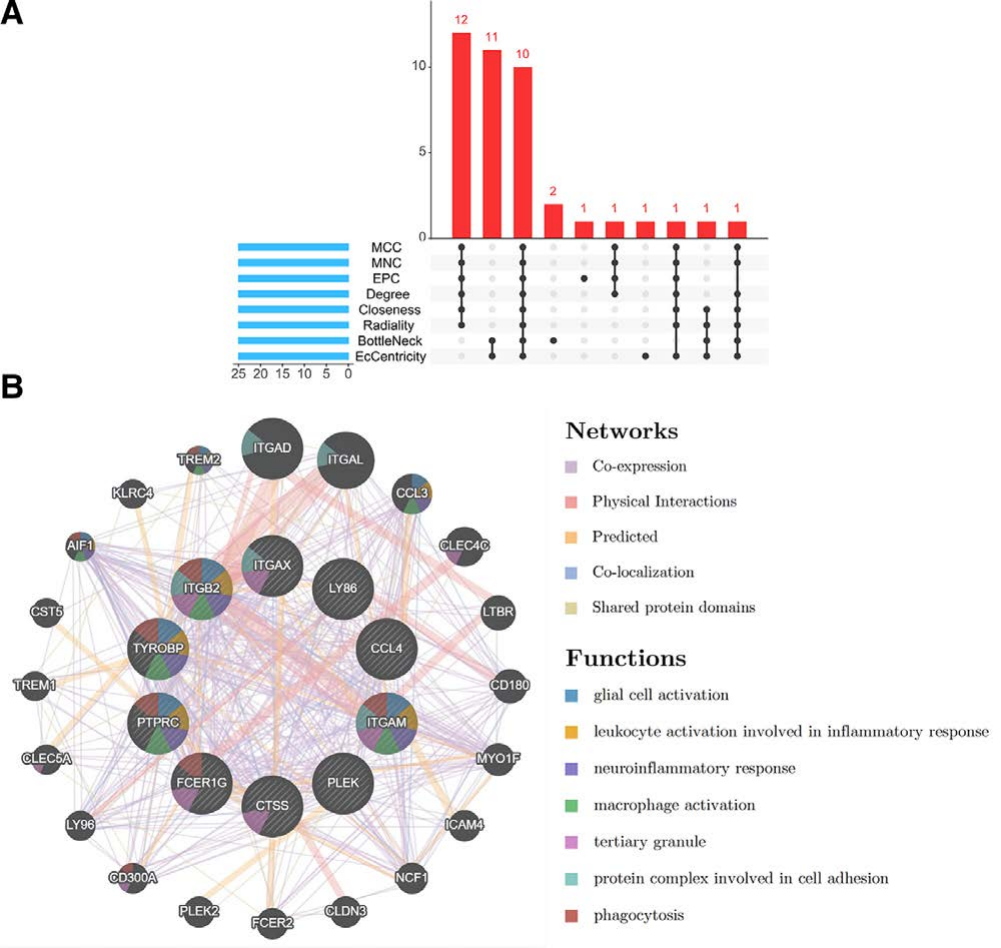
Identification of hub genes. (A) The upset plot showed that 8 algorithms have screened out 10 overlapping hub genes. (B) Hub genes and their co-expression genes were analysis by GeneMANIA.

**Figure 6.**
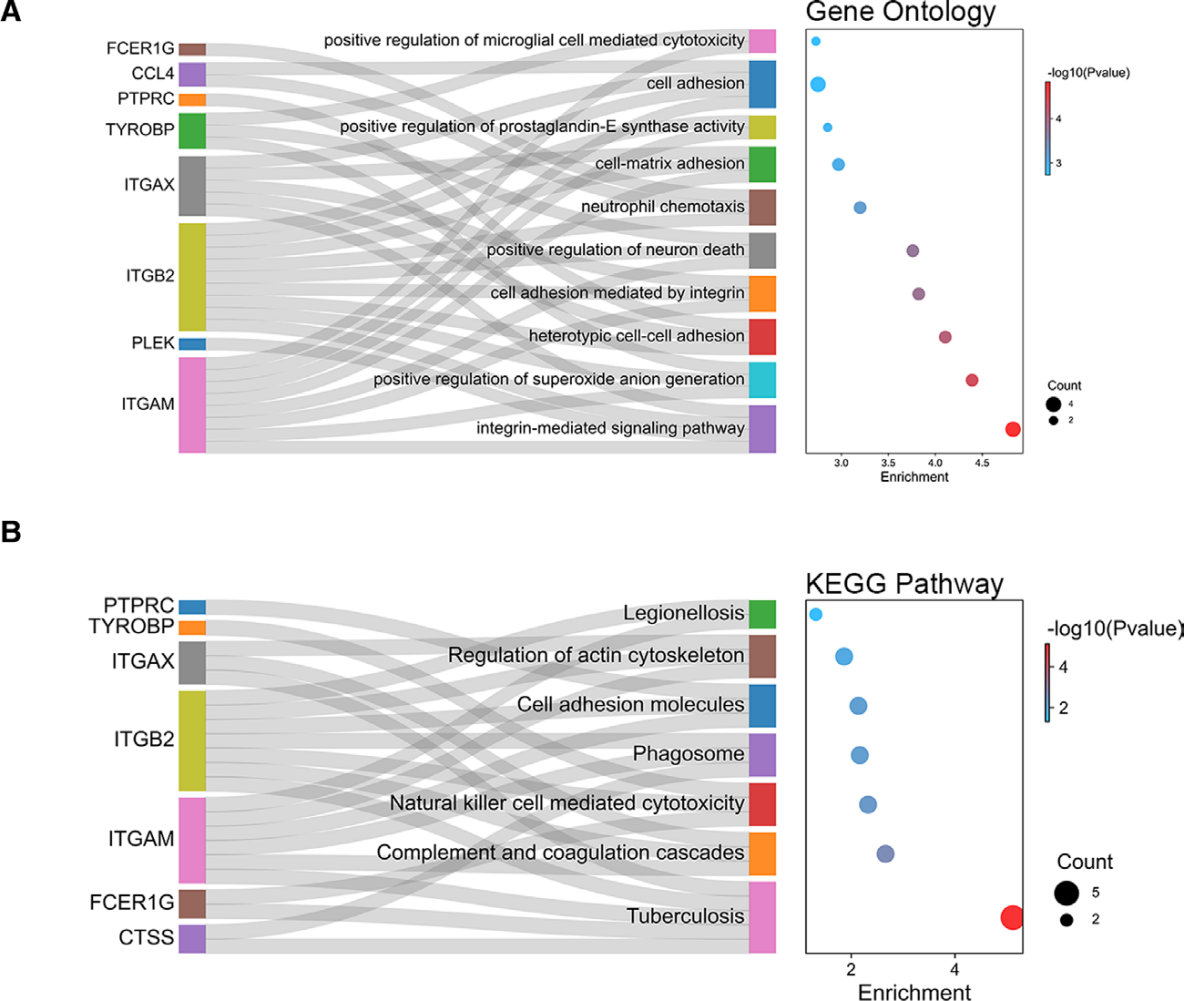
Enrichment analysis of the hub genes. (A) GO enrichment analysis of the hub genes. (B) KEGG Pathway enrichment analysis of the hub genes. GO = gene ontology; KEGG = Kyoto Encyclopedia of Genes and Genomes.

The top 25 hub genes were obtained using 8 plug-in cytoHubba algorithms (Table 1). Next, the 10 common hub genes were obtained using Upset diagrams: TYROBP, PTPRC, integrin subunit beta 2 (ITGB2), ITGAM, PLEK, cathepsin S (CTSS), lymphocyte antigen 86 (LY86), ITGAX, CCL4, and FCER1G (Fig. 5A; Table 2). The co-expression network and related functions of these hub genes, analyzed using the GeneMANIA database, revealed a complex PPI network with a co-expression of 58.88%, physical interactions of 29.53%, prediction of 6.00%, co-localization of 5.08%, and shared protein domains of 0.52% (Fig. 5B). These hub genes were closely involved in integrin-mediated signaling pathways, positive regulation of superoxide anion generation, cell adhesion mediated by integrin, neutrophil chemotaxis, and cell–matrix adhesion (Fig. 6A). These findings highlight the important role of integrins in atherosclerosis and AAA. In the KEGG pathway, complement and coagulation cascades, natural killer cell-mediated cytotoxicity, cell adhesion molecules, and regulation of the actin cytoskeleton were identified (Fig. 6B). This suggests that changes in ITGAM, ITGB2, and ITGAX may affect the actin cytoskeleton and promote formation of arterial aneurysms.

**Table 1:** The top 25 hub genes rank in cytoHubba.

MCC	MNC	EPC	Degree	Closeness	Radiality	Bottle neck	Eccentricity
TYROBP	TYROBP	TYROBP	TYROBP	TYROBP	TYROBP	PTPRC	APOE
PTPRC	PTPRC	PTPRC	PTPRC	PTPRC	PTPRC	TYROBP	ITGAM
ITGB2	ITGB2	ITGB2	ITGB2	ITGB2	ITGAM	APOE	CTSS
ITGAM	ITGAM	CD86	ITGAM	ITGAM	ITGB2	ITGB2	PTPRC
PLEK	PLEK	LCP2	PLEK	PLEK	PLEK	ITGAM	TYROBP
CD86	CD86	PLEK	CD86	CD86	CTSS	HLA-DRA	ITGB2
LCP2	LCP2	ITGAM	LCP2	LCP2	LCP2	MMP12	HLA-DRA
CTSS	CTSS	FCGR2B	CTSS	CTSS	TLR2	NCF2	MMP12
TLR2	TLR2	CSF1R	TLR2	TLR2	CD86	ITGAX	NCF2
CSF1R	CSF1R	CTSS	CSF1R	CSF1R	CSF1R	CD48	ITGAX
LAPTM5	LAPTM5	LAPTM5	LAPTM5	LAPTM5	LAPTM5	LCP1	CD48
C1QA	C1QA	IL10RA	C1QA	C1QA	C1QA	CORO1A	LCP1
CD53	IL10RA	TLR2	IL10RA	CD53	CD53	PLEK	CORO1A
IL10RA	CD53	HCK	CD53	IL10RA	AIF1	CTSS	PLEK
LY86	LY86	CD53	LY86	LY86	LY86	CXCR4	CXCR4
FCGR2B	FCGR2B	LY86	FCGR2B	FCGR2B	ITGAX	C1QC	C1QC
HCK	HCK	AIF1	HCK	HCK	IL10RA	SLAMF8	SCD
ITGAX	ITGAX	C1QA	ITGAX	ITGAX	FCGR2B	SCD	LY86
CCL4	CCL4	CCL4	CCL4	CCL4	HCK	AMPD3	C5AR1
FCER1G	FCER1G	FCER1G	FCER1G	FCER1G	CCL4	LY86	FCER1G
IRF8	IRF8	IRF8	IRF8	IRF8	IRF8	C5AR1	CCL4
AIF1	CCR1	ITGAX	CCR1	AIF1	C1QC	FCER1G	IGSF6
CCR1	CD48	C3AR1	CD48	CCR1	FCER1G	CCL4	CD52
CD48	AIF1	CD74	AIF1	CD48	CCR1	IGSF6	CD86
C3AR1	C3AR1	CCR1	C3AR1	C1QC	CD48	CD52	BCL2A1

**Table 2:** The details of the hub genes.

No.	Gene	Full name	Function
1	TYROBP	TYRO protein tyrosine kinase binding protein	This gene encodes a transmembrane signaling polypeptide which contains an immunoreceptor tyrosine-based activation motif (ITAM) in its cytoplasmic domain. The encoded protein may bind zeta-chain (TCR) associated protein kinase 70 kDa (ZAP-70) and spleen tyrosine kinase (SYK) and play a role in signal transduction, bone modeling, brain myelination, and inflammation.
2	PTPRC	Protein tyrosine phosphatase receptor type C	The protein encoded by this gene is a member of the protein tyrosine phosphatase (PTP) family. This PTP has been shown to be an essential regulator of T- and B-cell antigen receptor signaling. This PTP also suppresses JAK kinases.
3	ITGB2	Integrin subunit beta 2	This gene encodes an integrin beta chain, which combines with multiple different alpha chains to form different integrin heterodimers. Integrins are integral cell-surface proteins that participate in cell adhesion as well as cell-surface mediated signaling. The encoded protein plays an important role in immune response and defects in this gene cause leukocyte adhesion deficiency.
4	ITGAM	Integrin subunit alpha M	This gene encodes the integrin alpha M chain. This I-domain containing alpha integrin combines with the beta 2 chain (ITGB2) to form a leukocyte-specific integrin referred to as macrophage receptor 1 (“Mac-1”), or inactivated-C3b (iC3b) receptor 3 (“CR3”).The alpha M beta 2 integrin is important in the adherence of neutrophils and monocytes to stimulated endothelium, and also in the phagocytosis of complement coated particles.
5	PLEK	Pleckstrin	Involved in several processes, including G protein-coupled receptor signaling pathway; actin cytoskeleton organization; and positive regulation of supramolecular fiber organization.
6	CTSS	Cathepsin S	This gene participates in the degradation of antigenic proteins to peptides for presentation on MHC class II molecules. This gene is implicated in the pathology of many inflammatory and autoimmune diseases.
7	LY86	Lymphocyte antigen 86	Acts upstream of or within positive regulation of lipopolysaccharide-mediated signaling pathway.
8	ITGAX	Integrin subunit alpha X	This gene encodes the integrin alpha X-chain protein. The alpha X beta 2 complex seems to overlap the properties of the alpha M beta 2 integrin in the adherence of neutrophils and monocytes to stimulated endothelium cells, and in the phagocytosis of complement coated particles.
9	CCL4	C-C motif chemokine ligand 4	It is one of the major HIV-suppressive factors produced by CD8 + T cells. The encoded protein is secreted and has chemokinetic and inflammatory functions.
10	FCER1G	Fc epsilon receptor Ig	The high affinity IgE receptor is a key molecule involved in allergic reactions.

### 3.5. Validation of hub genes expression

**Figure 7.**
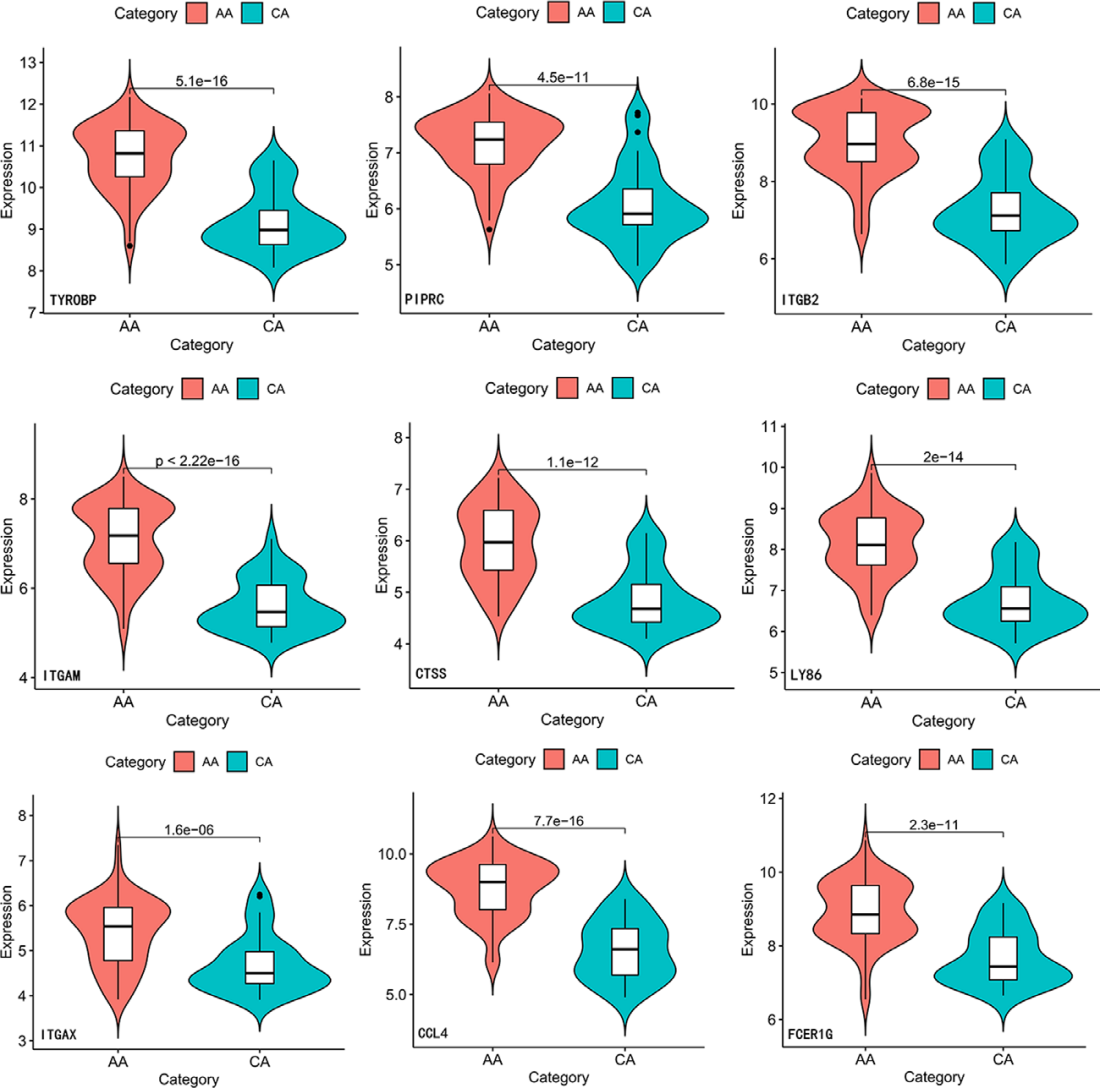
Verification of the expression level of hub genes in GSE100927. AA = atherosclerosis; CA = control artery.

**Figure 8.**
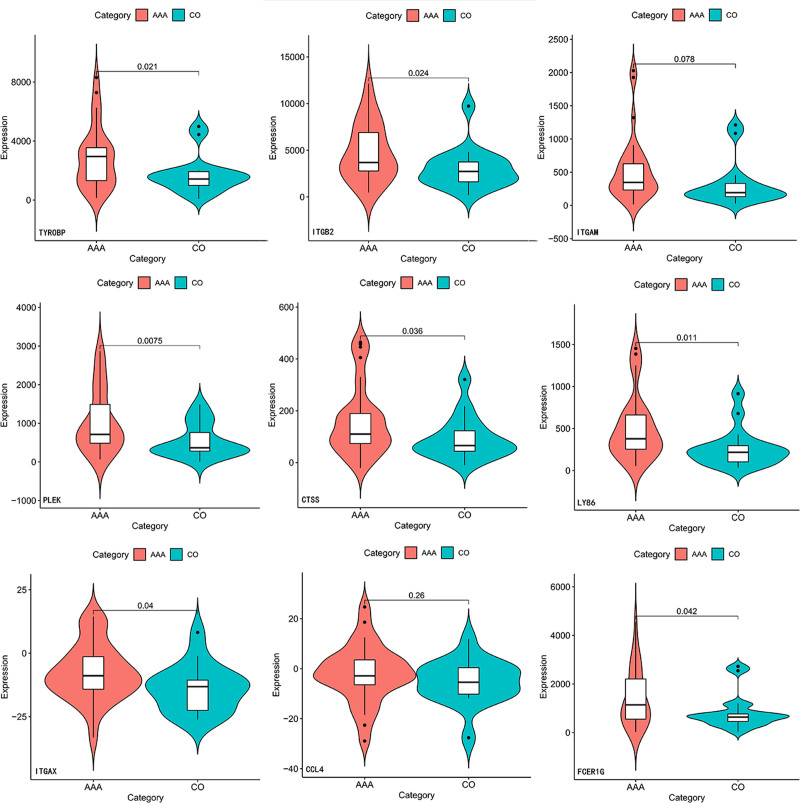
Verification of the expression level of hub genes in GSE98278. AAA = abdominal aortic aneurysms; CO = compared ruptured abdominal aortic aneurysms.

Two other datasets containing atherosclerotic plaques and AAAs were selected to confirm the reliability of these gene expression levels. In the GSE100927 dataset, the expression values of PLEK were missing, whereas the expression of other genes was upregulated in atherosclerotic plaques (Fig. 7). In the GSE98278 dataset, expression values of PTPRC were missing. In addition, expressions of ITGAM and CCL4 were not statistically significant. Expression of other genes was upregulated in AAA (Fig. 8). Combining the above results, TYROBP, ITGB2, CTSS, LY86, ITGAX, and FCER1G were expressed in these datasets with the same tendency as in the original datasets.

### 3.6. Prediction and verification of TFs

**Figure 9.**
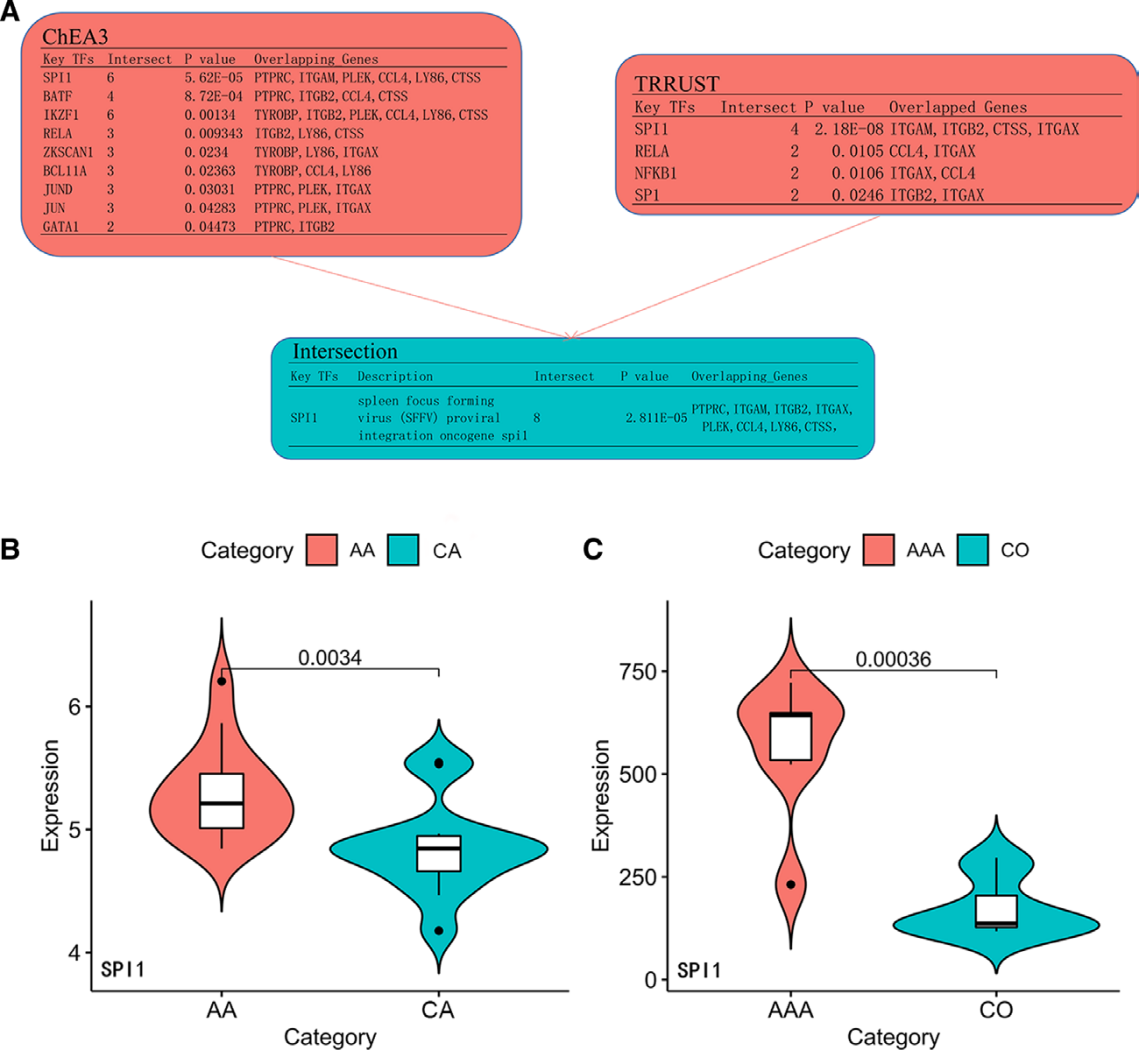
Predicting the results and the expression level of TFs. (A) Predicting the results of TFs in ChEA3 database and TRRUST database respectively and the intersection of 2 databases. (B) The expression level of TF expression was verified in GSE28829. (C) The expression level of TF expression was verified in GSE7084. ChEA3 = ChIP-X enrichment analysis 3; TF, transcription factor; TRRUST = Transcriptional Regulatory Relationships Unraveled by Sentence-based Text mining.

Using the ChEA3 database, 9 TFs were predicted to regulate the expression of these hub genes, with 4 TFs predicted to regulate the expression of these hub genes, using the TRRUST database. Of these, only 1 transcription factor (SPI1) was common to both databases (Fig. 9A) and shown to be highly expressed in both diseases (Fig. 9B and C), being involved in the regulation of 8 hub genes (PTPRC, ITGAM, ITGB2, ITGAX, PLEK, CCL4, LY86, and CTSS).

## 4. Discussion

Our identification of common hub genes for atherosclerosis and AAA provides new insights into the shared biological mechanisms of these 2 diseases. Our findings of an association between atherosclerosis and AAA is consistent with previous studies.^[21,22]^ Identification of the common DEGs for atherosclerosis and AAA will help to explore their common pathogenesis, identify new therapeutic targets, and predict the therapeutic effect of biological agents.

Our study identified 133 overlapping DEGs between atherosclerosis and AAA, including 10 hub genes (TYROBP, PTPRC, ITGB2, ITGAM, PLEK, CTSS, LY86, ITGAX, CCL4, and FCER1G). GO and KEGG pathway enrichment analyses showed that these genes were involved in integrin-mediated signaling pathways, integrin-mediated cell adhesion, neutrophil chemotaxis, regulation of the actin cytoskeleton, chemokine signaling pathways, and antigen processing and presentation. These results demonstrate the important role of integrins, chemokines, and immune and inflammatory responses in both diseases. The GO analysis identified that integrin-mediated signaling pathways play an important role in both diseases. Furthermore, that leukocyte integrin αxβ2 was upregulated under hypercholesterolemic conditions with reduced atherogenesis after its deletion suggests that αxβ2 may be particularly important in atherogenicity.^[23]^ Deposition of matrix proteins in atherosclerotic plaques creates a permissive environment for cell proliferation, migration, differentiation, and inflammatory responses, primarily via integrin α5β1 and αvβ3 signaling.^[24]^ Fibroblast growth factor 18 and integrin β1 can improve the repair of AAA by increasing elastin expression, enhancing the migration and proliferation of smooth muscle cells, and improving aortic remodeling.^[25]^ Therefore, integrins may be the link between atherosclerosis and AAA.

In our study, we further identified 9 TFs in the TRRSUT database and 4 TFs in the ChEA3 database which may regulate the expression of the identified hub genes. By combining these results, the high reliability of expression of 1 TF (SPI1) in atherosclerosis and AAA was confirmed. SPI1 is involved in the regulation of 8 hub genes (PTPRC, ITGAM, ITGB2, ITGAX, PLEK, CCL4, LY86, and CTSS). Of these, after gene expression verification, only ITGB2, CTSS, LY86, and ITGAX were found to be highly expressed in both atherosclerosis and AAA.

ITGB2 encodes the integrin beta chain. The protein encoded by this gene plays an important role in immune responses, with a defect of this gene leading to defective leukocyte adhesion. ICAM1 and endothelial cells recruit circulating ITGB2, also known as CD18, and immune cells contribute to atherosclerosis; therefore, inhibition of ITGB2 can alleviate or even prevent the development of atherosclerosis.^[26]^ Animal experiments have shown that treatment of mice with AAA using an anti-CD18 monoclonal antibody alleviates AAA expansion and reduces the inflammatory response,^[27]^ indicative of the potential benefit of ITGB2 downregulation in patients with AAA.

CTSS is a lysosomal cysteine proteinase that participates in the degradation of antigenic proteins into peptides for presentation on MHC class II molecules. CTSS is involved in the pathogenesis of cardiovascular diseases via its effect on extracellular matrix protein degradation, protein transport, and cell signaling.^[28]^ CTSS can be secreted into the extracellular matrix via lysosomes, increasing collagen and elastin degradation, promoting vascular smooth muscle migration, and ultimately causing atherosclerosis.^[29]^ Apoptosis of the medial smooth muscle cells of the arterial wall is an important marker of AAA, with an increase in apoptosis during aneurysm formation. Reduction of CTSS has been shown to attenuate smooth muscle cell apoptosis in the aorta, in vitro and reduce smooth muscle cell loss in AAA lesions.^[30]^

LY86, also known as MD-1, can form a complex with RP105 to block the TLR4/MD-2 complex and, thus, attenuate inflammation via the NF-KB signaling pathway.^[31]^ Therefore, an RP105 deficiency can lead to a slower progression of early atherosclerotic plaques.^[32]^ Divanovic et al showed that RP105 can suppress TLR4 signaling only when MD-1 is fully present.^[33]^ Therefore, the detailed mechanism by which the specific RP105/MD-1 complex leads to atherosclerosis needs to be further elucidated. The expression of LY86 was not limited to immune cells but was also highly expressed in cardiovascular tissues. LY86 plays an important role in cardiac remodeling, myocardial hypertrophy, fibrosis, arrhythmia, and heart failure.^[34]^ Although the effect of LY86 on AAA is still unclear, we believe that LY86 also plays an important role in the pathogenesis of AAA.

Integrin subunit alpha X (ITGAX), known as CD11C, encodes an integrin X-chain protein that binds to ITGB2 to form a leukocyte-specific integrin called inactivated-C3b (iC3b) receptor 4 (CR4). ITGAX is a fibrinogen receptor that is important for monocyte adhesion and chemotaxis, which mediates cell-to-cell interactions during inflammatory responses. Monocytes are among the main cells involved in atherosclerosis. ITGAX can mediate the adhesion of monocytes to endothelial cells and then infiltrate the arterial wall through endothelial cells,^[35]^ which is an important link in the formation of atherosclerosis.^[36]^ In addition, CD11C expression in macrophages is regulated by interferon regulatory factor-5, promoting the presence of CD11C-expressing macrophages within atheromatous plaques.^[37]^ Previous studies have shown that CD4 T cells and CD8 + T cells decrease significantly after CD11C deletion, which can further down-regulate activity of neutrophil elastase, thus decreasing elastase degradation and increasing collagen content and, overall, inhibiting degradation of the abdominal aortic matrix.^[38]^ However, few studies have explored the relationship between atherosclerosis and AAA.

Our study focused on the common hub genes and related transcription factors in atherosclerosis and AAA. Hub genes were identified using a complex network of interactions and key nodes. This bioinformatics approach has proven to be reliable for other diseases.^[39–41]^ Moreover, we verified the expression levels of the hub genes and transcription factors, which made our results more credible. We believe that our results provide a new research direction for the molecular mechanism of atherosclerosis complicated by AAA.

The limitations of our study need to be acknowledged. The datasets we selected were from different platforms and, therefore, the detection methods and algorithms for the platforms are bound to be different. In the future, we plan to use a microarray from the same platform to test our patients to eliminate this difference. The function of hub genes also needs to be further verified in cell and animal models, which will be the focus of our future studies. In future studies, we suspected that it may be possible to detect hub genes expression levels in the blood of patients with 2 diseases to predict the trend of disease occurrence.

## 5. Conclusions

Common DEGs associated with atherosclerosis and AAA were identified and subjected to enrichment analysis and PPI network analysis, identifying a common pathogenetic pathway which may be mediated by specific hub genes. Specifically, we identified that integrin-related genes may play significant roles in both diseases. Our findings may provide new directions for research on the molecular mechanisms of atherosclerosis complicated by AAA.

## Author contributions

**Conceptualization:** Degao Hong, Likang Ma.

**Data curation:** Degao Hong, Likang Ma.

**Formal analysis:** Degao Hong, Likang Ma.

**Funding acquisition:** Zhihuang Qiu.

**Investigation:** Likang Ma, Lei Jin.

**Methodology:** Lei Jin.

**Validation:** Lele Tang.

**Visualization:** Lele Tang.

**Writing – original draft:** Degao Hong, Likang Ma.

**Writing – review & editing:** Liangwan Chen, Zhihuang Qiu.

## Supplementary Material
