# Supplementary material for: The role of integrin-related genes in atherosclerosis complicated by abdominal aortic aneurysm
Source: Medicine (Baltimore). 2024 Nov 15;103(46):e40293. doi: 10.1097/MD.0000000000040293 (PMC11576009; doi:10.1097/MD.0000000000040293)
Supplement: Supplementary file 1 [file medi-103-e40293-s001.docx]

**S-TABLE 1: The expression levels of the DEGs.**

| **Gene Symbol** | **Expression** | **GSE28829 Log2(FC)** | **GSE7084 Log2(FC)** |
| --- | --- | --- | --- |
| VAMP8 | U | 1.82538528 | 2.3 |
| CXCR4 | U | 2.01558108 | 1.78 |
| CYBA | U | 1.17000376 | 2.51 |
| C1QC | U | 1.77348421 | 1.52 |
| C15orf48 | U | 1.36449082 | 2.35 |
| SLC2A5 | U | 1.06111853 | 1.58 |
| HLA-DRB4 | U | 1.68511663 | 2.46 |
| AQP9 | U | 1.56843121 | 1.99 |
| DOCK2 | U | 1.16525063 | 1.99 |
| FPR3 | U | 1.52700769 | 2.04 |
| ALOX5 | U | 1.8734971 | 1.98 |
| PLEK | U | 1.35108993 | 2.7 |
| RNASE6 | U | 1.53279991 | 1.29 |
| FCGR2B | U | 2.04459216 | 1.68 |
| C5AR1 | U | 1.31277311 | 1.8 |
| ARHGAP30 | U | 1.1716834 | 1.92 |
| CNN1 | D | -1.08205995 | -2.88 |
| RAC2 | U | 1.54707589 | 2.17 |
| ADAMDEC1 | U | 1.79015611 | 1.68 |
| SEL1L3 | U | 1.40422097 | 1.83 |
| APOE | U | 2.19024169 | 2.87 |
| FABP5 | U | 1.65414816 | 1.83 |
| SYNC | D | -1.05038478 | -1.49 |
| C3AR1 | U | 1.59008322 | 1.31 |
| NCF2 | U | 1.5377853 | 2.2 |
| PDZRN3 | D | -1.09055942 | -1.22 |
| HLA-DRA | U | 1.41308569 | 2.18 |
| BAMBI | D | -1.08334888 | -1.59 |
| LY86 | U | 1.59914143 | 2.14 |
| GMFG | U | 1.05534996 | 1.81 |
| ACADL | D | -1.36528677 | -1.75 |
| FERMT3 | U | 1.10670507 | 1.79 |
| CD53 | U | 1.66446979 | 2.56 |
| SERPINA1 | U | 1.73250228 | 2.3 |
| SNX10 | U | 1.2093328 | 2.18 |
| MS4A6A | U | 1.23529239 | 1.73 |
| HCK | U | 1.42467088 | 1.44 |
| IER3 | U | 1.85335214 | 1.59 |
| CTSB | U | 1.31096558 | 1.55 |
| CTSS | U | 1.78370797 | 2.24 |
| AIM1 | U | 1.24861532 | 1.79 |
| HLA-DPA1 | U | 1.33154415 | 1.31 |
| CPVL | U | 1.49159451 | 1.95 |
| SGK1 | U | 1.29262713 | 2.5 |
| HLA-DMB | U | 1.46503124 | 2.13 |
| SCD | U | 1.55544016 | 1.83 |
| KYNU | U | 2.03619731 | 1.68 |
| CAPG | U | 1.42516773 | 2.38 |
| SLAMF8 | U | 2.07254568 | 1.88 |
| LIPA | U | 1.2261902 | 1.6 |
| CD83 | U | 1.06179492 | 3.02 |
| SMPDL3A | U | 1.06746747 | 1.09 |
| CCL4 | U | 1.09394133 | 3.15 |
| ITGB2 | U | 2.03750182 | 2.05 |
| PRUNE2 | D | -1.08904506 | -2.63 |
| AIF1 | U | 1.36579748 | 1.75 |
| THEMIS2 | U | 1.09259043 | 2.23 |
| CD86 | U | 1.88105176 | 1.88 |
| TNFAIP3 | U | 1.12780089 | 1.41 |
| AMPD3 | U | 1.25375757 | 1.5 |
| MMP12 | U | 2.57619745 | 2.68 |
| CARD16 | U | 1.12324036 | 1.03 |
| REEP1 | D | -1.25231484 | -1.12 |
| BCL2A1 | U | 1.40446315 | 1.66 |
| ANGPTL1 | D | -1.15015863 | -1.05 |
| UCP2 | U | 1.19214615 | 2.59 |
| PTPRC | U | 1.55945298 | 1.72 |
| CD14 | U | 1.69607315 | 2.15 |
| ITGAM | U | 1.50837718 | 1.52 |
| CD37 | U | 1.51164178 | 2.74 |
| FCGR2A | U | 1.33960174 | 1.08 |
| TYROBP | U | 1.6977148 | 2.56 |
| CCR1 | U | 1.96545736 | 2.2 |
| ATP1A2 | D | -1.83391996 | -1.62 |
| LYN | U | 1.14745246 | 1.95 |
| ACP5 | U | 2.05226723 | 2.48 |
| GPR183 | U | 1.12522343 | 2.27 |
| ALOX5AP | U | 1.29445279 | 1.49 |
| PIK3CG | U | 1.10231597 | 1.47 |
| CXCL2 | U | 2.0343571 | 2.5 |
| CCL8 | U | 1.14189585 | 2.6 |
| TNFSF13B | U | 2.18450919 | 1.69 |
| CEMIP | U | 1.71136881 | 1.96 |
| CD74 | U | 1.08725697 | 1.52 |
| CORO1A | U | 1.11759114 | 2.42 |
| EVI2B | U | 1.66658113 | 2.05 |
| CD52 | U | 1.57745511 | 3.31 |
| BAG2 | D | -1.20934135 | -1.49 |
| PNMAL1 | D | -1.11006147 | -1.2 |
| NEXN | D | -1.05580843 | -2.31 |
| CYTIP | U | 1.42081248 | 1.54 |
| C2 | U | 1.25626406 | 1.52 |
| FBP1 | U | 1.17443752 | 2.46 |
| PLA2G7 | U | 1.20407721 | 2.03 |
| ANPEP | U | 1.20073886 | 1.76 |
| CD48 | U | 1.32341846 | 2.33 |
| ITGAX | U | 1.07830597 | 2.16 |
| IL1RN | U | 1.27257462 | 2.79 |
| APCDD1 | D | -1.02873894 | -1.12 |
| MS4A7 | U | 1.68468145 | 1.3 |
| LCP2 | U | 1.20091101 | 2.03 |
| IL10RA | U | 1.01958141 | 1.67 |
| RNASE1 | U | 1.42335929 | 1.64 |
| ADAP2 | U | 1.22371715 | 1.85 |
| CCL5 | U | 1.08731108 | 2.13 |
| HLA-DMA | U | 1.48883125 | 1.89 |
| MYO1F | U | 1.1116221 | 1.4 |
| CD84 | U | 2.07858481 | 1.6 |
| CLEC5A | U | 1.53990541 | 1.64 |
| LAPTM5 | U | 1.58468391 | 2.33 |
| SBSPON | D | -1.01643072 | -1.66 |
| LCP1 | U | 1.24965701 | 2.55 |
| MPEG1 | U | 1.2971224 | 2.49 |
| SH3BGR | D | -1.1703176 | -1.92 |
| IRF8 | U | 1.12706498 | 1.62 |
| PDE8B | D | -1.17153489 | -2.06 |
| MARCO | U | 1.20119944 | 1.57 |
| IGSF6 | U | 1.07902249 | 1.7 |
| FCER1G | U | 1.49918477 | 1.61 |
| C1QA | U | 1.45294564 | 1.26 |
| TREM1 | U | 1.41186934 | 1.93 |
| CASQ2 | D | -1.16446042 | -1.79 |
| TLR2 | U | 1.07309979 | 1.23 |
| CSF1R | U | 1.17245573 | 2.04 |
| CXCL16 | U | 1.37661328 | 1.65 |
| NPL | U | 1.91658872 | 1.57 |
| DENND2D | U | 1.15465237 | 1.43 |
| DAPK1 | U | 1.23474996 | 1.71 |
| APOC1 | U | 1.82242297 | 2.8 |
| TMEM176B | U | 1.36009518 | 1.08 |
| HCLS1 | U | 1.05908364 | 2.05 |
| MMP9 | U | 2.01113047 | 3.99 |
| CTSC | U | 1.29911402 | 1.24 |
